# State of the Art Progress in Copper Vanadate Materials for Solar Water Splitting

**DOI:** 10.3390/nano13182599

**Published:** 2023-09-20

**Authors:** Shankara S. Kalanur, Jaldappagari Seetharamappa, Qadeer Akbar Sial, Bruno G. Pollet

**Affiliations:** 1 Green Hydrogen Lab (GH2Lab), Institute for Hydrogen Research (IHR), Université du Québec à Trois-Rivières (UQTR), 3351 Boulevard des Forges, Trois-Rivières, QC G9A 5H7, Canada; 2 Department of Chemistry, Karnatak University, Dharwad 580003, India; drjseetharamappa@kud.ac.in; 3 Department of Advanced Materials Chemistry, Korea University, Sejong 339-700, Republic of Korea; qadeersial@ajou.ac.kr

**Keywords:** photoelectrochemical water splitting, CuV_2_O_6_, Cu_2_V_2_O_7_, Cu_3_V_2_O_8_, Cu_5_V_2_O_10_, Cu_11_V_6_O_26_

## Abstract

The development of a single junction photoelectrode material having specific properties is essential and challenging for the efficient application in solar water splitting for oxygen production and a high value-added product, hydrogen. Moreover, the present material solutions based on binary metal oxides offer limited catalytic activity and hydrogen production efficiency. Therefore, it is paramount to develop and exploit a unique range of materials derived from ternary metal oxides with specifically engineered properties to advance in photoelectrochemical (PEC) water splitting. Among the ternary oxides, copper vanadates offer promising characteristics, such as a narrow bandgap and catalytic surface properties along with favorable band edges for facile oxygen evolution reaction (OER), which is considered the bottleneck step in performing overall water dissociation. Furthermore, the copper vanadates allow the tuning of the stoichiometry through which a wide range of polymorphs and materials could be obtained. This review provides a complete outlook on the range of copper vanadates and the established synthesis approach, morphology, crystal structure, band edge properties, and PEC characterizations. Mainly, the underlying charge dynamic properties, carrier path length, effect of doping, and influence of surface catalysts are discussed. The review concludes that the advancement toward obtaining low-bandgap materials is a main challenge to overcome the limitations for efficient water dissociation to OER and copper vanadates, which offer a promising solution with their unique properties and advantages. Importantly, intense and strategically focused research is vital to overcome the scientific challenges involved in copper vanadates and to explore and exploit new polymorphs to set new efficiency benchmarks and PEC water splitting solutions.

## 1. Introduction

The widespread consumption of fossil fuels that strengthened the rapid evolution of the human lifestyle, technological development, modernization, and industrialization in the last century have implicated the significance and role of energy sources for future advancement [1,2]. Specifically, fossil fuels are at the forefront of such an extraordinary advancement until now. Hence, fossil fuels have been portrayed as an ideal source of energy due to their ease of extraction, refining, accessibility, and the cost that leads to their extensive consumption [3]. However, despite having profound advantages, the extensive use of fossil fuels is causing several irreversible environmental issues via global warming, which are connected to carbon dioxide (CO_2_) emissions [4]. The unprecedented natural catastrophes that occurred in the last decade due to greenhouse gas emissions have forced researchers to explore and shift energy dependency toward environment-friendly, renewable, and sustainable routes to support human advancement without causing damage to the environment [5,6]. Hence, the urgent transformation to clean energy technology has become essential and a prime research focus in the present and in the future.

Concerning the sustainable and renewable energy carriers, the last few decades have witnessed a profound transformation to clean energy production technologies, such as solar photovoltaics, wind power, biofuels, etc. [7,8] Even though the transformation is taking place steadily toward sustainable and renewable techniques, their dependency on external/environmental factors (such as weather, available wind, water, or sunlight) and storage of surplus energy has become a major limitation. This has led to the further exploration of effective energy sources that offer the feasibility of storage, transportation, and utilization both in remote places either by small-scale or large-scale productions. Interestingly, in the last decade, energy storage systems based on lithium (lithium batteries) have advanced immensely toward dominating vehicles/transportation and logistics sectors, thereby neutralizing, to a minor extent, the effect of greenhouse emissions on the environment [9,10,11]. Even though the transformation toward electric vehicles is taking place rapidly, the utilization of lithium still faces several disadvantages [12]. That is, lithium ion is less abundant, expensive, and difficult to recycle indicating the further need of exploring alternate energy carriers or storage technologies. Moreover, lithium-based energy storage materials suffer from leakages and poor cycle stability, and are unsuitable for energy storage in the long term [12]. Conclusively, the worldwide transition toward a carbon-neutral society demands a significant revolution in energy production, transportation, storage, and utilization infrastructure.

## 2. Hydrogen

**Figure 1:**
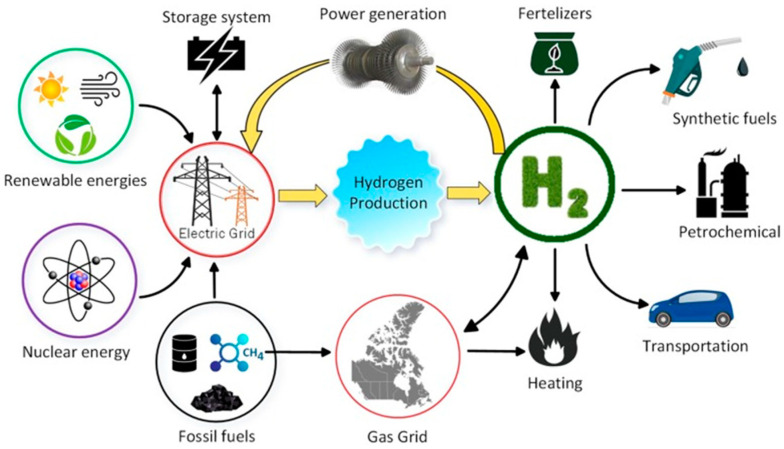
The applications of hydrogen and its production pathways. Reproduced with permission from [14] Elsevier, 2020.

Among the currently available energy carriers, hydrogen (H_2_) is the ideal substitute for carbon-based power sources due to its abundance and highly specific energy density [13]. Figure 1 represents the major consumers of hydrogen as an energy source for transportation, heating, and synthetic fuels in fertilizers and petrochemical industries [14]. Importantly, H_2_ is a promising source that could accelerate the advancement of renewable and sustainable energy infrastructure [15]. Using fuel cell technology, hydrogen can be readily used to generate electricity in both mobile and stationary systems either in small- or large-scale units [16].

Fuel cell systems produce only water as its byproducts from the electricity generation process after combining with oxygen, offering pure carbon-free green technology in a wide range of applications. Moreover, the energy per unit mass produced by hydrogen is three times more than that generated by the combustion of gasoline [17]. Due to its wider extraction sources (sources from which hydrogen could be produced), the efficient production industry could potentially boost the economy of the country and lift the energy dependency from outsourcing. Due to its atomic presence in a wide range of materials, hydrogen could be potentially extracted via sewage sludge, biofuels, gas, oil, and other hydrogen-containing resources.

## 3. Hydrogen Production Pathways

Hydrogen production technologies have been gaining significant interest in the last few decades as their demand has substantially increased. At present, about ~80% of its consumption is directed to fertilizer and petroleum industries and is expected to increase further as the utilization of hydrogen in transportation and power generation (houses) is gaining traction, considering its greener nature. Hydrogen is generally derived through a wide range of sources, including biomass, water, fossil fuels, hydrogen sulfide, etc., and through different methodologies which have several advantages and disadvantages (Figure 1). The feedstocks of hydrogen are classified into either renewable or non-renewable based on their origins/sources and environmental impacts (Figure 1). Due to the increasing demand for hydrogen, production using renewable and sustainable sources is recommended; this could provide economic independence and fulfil demands, along with providing environmental safety. However, among the currently available technologies, the production of hydrogen via fossil fuels is considered to be more economically viable despite posing a negative impact on the environment due to greenhouse gas emissions. That is, the hydrogen produced via fossil fuel reforming such as auto-thermal, partial, and steam methane reforming (SMR) are economically viable compared to the other technologies. However, new norms in carbon emission and commitment to limit the carbon footprint by most countries indicate the need for sustainable and greener hydrogen production technology. Moreover, without an efficient and economically viable carbon capture and conversion infrastructure, SMR technology faces an unpredictable future. Therefore, development toward greener and more sustainable hydrogen production technologies using renewable resources is encouraged to fulfil the hydrogen demand with strong economic growth and independence.

The electrolysis/splitting of water under the influence of applied potential is a well-known technique of hydrogen production discovered in the early 1800s, which was further developed, improvised, and optimized extensively for enhanced efficiency in recent decades. Considering its availability, H_2_O is the most abundant source through which hydrogen could be extracted. Mainly, the water in the oceans could potentially offer an abundant source of hydrogen through water electrolysis. Therefore, the electrolysis of water using abundant water sources and renewable energies with cost-effective components is considered the ideal strategy for an efficient and pilot-scale production of hydrogen in the future. At present, alkaline water electrolysis (AWE) and proton exchange membrane water electrolysis (PEMWE) techniques have demonstrated industrial-scale production of H_2_ with significant stability and efficiency and are commercially established in industries. Even though techniques such as anion exchange membrane water electrolysis (AEMWE) and solid oxide electrolysis cell (SOEC) are exhibiting improved hydrogen production efficiency and stability, their commercial prospectus requires significant R&D to compete with AWE and PEMWE technologies. Despite commercial success, the AWE and PEMWE technologies could not compete with the current market-dominated SMR technologies in terms of cost and production capability, indicating the necessity of significant improvement in electrolysis technologies.

Despite the commercial capability and productivity, AWE and PEMWE technologies still face limitations and challenges. That is, the water splitting thermodynamics in AWE require high overpotential and highly basic conditions that affect both costs of energy input and competent stability and lifetime issues, whereas the acidic conditions and use of precious metals in PEMWE result in high infrastructural and maintenance costs affecting commercial hydrogen availability. Therefore, exploiting new and optimizing different electrolysis techniques is essential to achieve the energy demand considering environmental and cost factors of renewable technologies [18].

## 4. Photoelectrochemical Water Spitting

The overpotential required to split water into O_2_ and H_2_ can be minimized using semiconductor photoelectrodes in the electrolysis setup with light illumination that provides significantly energetic photoexcited electrons and holes to produce hydrogen and oxygen, respectively, at lower overpotential—which is termed as photoelectrochemical (PEC) water splitting. Fujishima and Honda first reported the fragmentation of water to H_2_ and O_2_ using a semiconductor (TiO_2_) under UV light in a photocatalytic setup when performing water splitting [19]. Later, the photocatalytic technique was exploited in an electrochemical cell to assist the solar water splitting process with limited overpotential using a wide range of semiconductors [20]. Due to its advantages over electrolysis, the photocatalytic/PEC water splitting technique is considered as the ideal pathway for H_2_ production [21].

**Figure 2:**
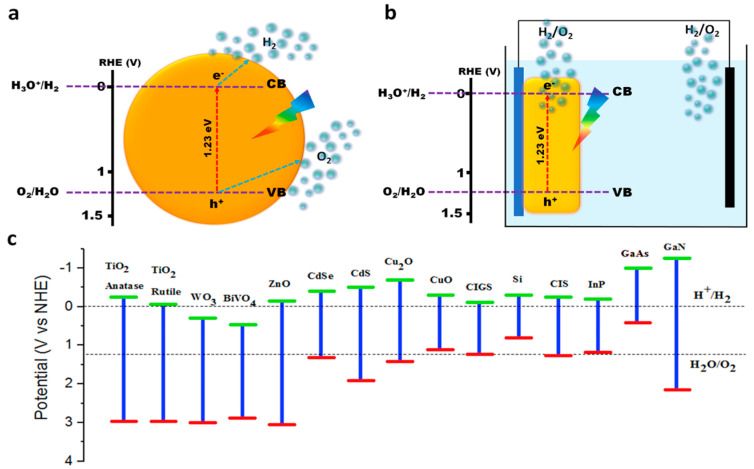
Working mechanism of photocatalytic (**a**) and PEC water splitting (**b**) under illumination and immersed in aqueous solution/electrolytes. (**c**) Band edge positions of largely used semiconductors plotted vs. NHE potentials for photocatalytic/PEC water splitting applications. Reproduced with permission from [22] Elsevier, 2017.

Figure 2 shows the mechanism of the water splitting process in both the photocatalytic and PEC modes using semiconductors under the influence of illuminated light immersed in aqueous solution/electrolytes. Principally, during the photocatalytic water splitting, the semiconductor absorbs photons (preferably in the visible region) to produce photoexcited electrons and holes which are diffused to the surface of the particle to catalytically react with water to generate O_2_ and H_2_, respectively. Alternatively, in PEC water splitting, the semiconductors are connected as electrodes in an electrochemical cell with aqueous electrolytes producing photoelectrons and holes during the light illumination, which are driven to the surface/circuit with the application of a bias potential to produce both H_2_ and O_2_ at cathode and anode electrode surfaces, respectively, based on n or p-type characteristics [22]. For efficient photocatalytic/PEC water splitting, the semiconductors are required to possess several properties such as narrow bandgap, ideal conduction band (CB)/valence band (VB) position, stability, surface catalytic, and efficient charge transfer/diffusion properties. That is, the semiconductor should possess a narrow bandgap (ideally 1.23 eV < bandgap < 3 eV) to extract a significant quantity of photons within the visible region to effectively utilize sunlight. In addition, the CB positions must be located above (have more negative potential than) 0 V vs. RHE (H_2_ production potential), whereas the VB must be below the 1.23 V vs. RHE (water oxidation potential) to ideally utilize the photoexcited electrons and holes to reduce and oxidize water to H_2_ and O_2_, respectively (Figure 2a,b). To defuse/transfer the photoexcited charges toward the semiconductor surface, the materials should have an effective carrier path length/conductivity to avoid recombination and effective consumption of photoelectrons and holes. Finally, the surface of the semiconductor should possess catalytic properties and stability to utilize the photoexcited holes and electrons to generate O_2_ and H_2_, respectively, without degrading/oxidizing/leaching (photocorrosion) the semiconductor materials. Conclusively, the ideal material has to satisfy the aforementioned requirements to produce hydrogen and oxygen efficiency using sunlight. However, experimentally driving such a reaction in the photocatalytic/PEC cell faces several challenges that hinder the overall efficiency due to the semiconductor’s property limitations and stability.

Given the material property requirements, an extensive array of materials are explored and exploited for photocatalytic and PEC experiments. Figure 2c represents the CB and VB edge potential and bandgap of largely employed materials in solar water splitting applications [23]. Among the materials, the rutile and anatase phase of TiO_2_ and ZnO has been widely employed for solar water splitting applications, owing this to their suitable CB/VB locations concerning overall water splitting potential [24,25], as shown in Figure 2c. However, due to the wider bandgap, the absorption range of TiO_2_ falls in the UV region of the incident light [26]. Moreover, the surface of TiO_2_ is inefficient in utilizing photogenerated holes and thus requires a surface catalyst and a visible light sensitizer to perform an oxygen evolution reaction and absorb the visible region of the light [27,28]. The WO_3_ [29] and BiVO_4_ [30,31] have demonstrated excellent oxygen evolution capabilities under the irradiation conditions; however, the presence of their conduction band edge below water reduction potential does not allow facile hydrogen production using photogenerated electrons. The BiVO_4_ suffers from photocorrosion due to V leaching, whereas the WO_3_ is relatively unstable in neutral pH and corrodes in basic conditions. Furthermore, the BiVO_4_ surface shows poor catalytic activity toward the OER reaction, while WO_3_ absorbs the limited visible light due to the wide bandgap compared to that of BiVO_4_.

Similarly, several n-type (CdS, CdSe) [32,33] and p-type (CIS, CIGS) [34,35] chalcogenides, nitrides (TaN, GaN) [36,37], phosphides (InP) [38], and Cu_2_Os [39] have been explored for solar water splitting applications, owing this to their narrow bandgap that absorbs sufficient visible light and well-matched band edge positions concerning overall water splitting potential. However, these materials suffer from poor stability and thus require catalytic support, coatings for improved stability, and long-term operation [38,39,40,41,42,43]. On the other hand, several materials such as Si, CuO, GaAs, etc. have demonstrated excellent H_2_ production capabilities due to their narrow bandgaps that absorb a significant portion of visible light and due to the suitability of their conduction band edge position. However, due to their unfavorable valence band edge position situated significantly above water oxidation potential provides energetically ineffective holes to oxidize water for O_2_ production [44,45,46]. Moreover, the utilization of such photocathodes is limited due to the poor charge diffusion/catalysis, stability, and toxic nature, respectively [45,46,47]. In addition, several strategies have been used to enhance the PEC properties of semiconductors, which include catalyst coating [48], doping [49], oxygen vacancy engineering [50], work function tuning [51], heterojunction formation [52], etc. Convincingly, the comprehensive and extensive research progress from the literature implicates that the utilization of suitable materials provides the key to efficient and stable solar water splitting reactions. Moreover, considering the green and sustainable nature of the PEC water splitting technology, exploiting new materials is essential, which poses a significant challenge for the future.

## 5. Copper Vanadates

Metal vanadates (MVs) have demonstrated promising photocatalytic and PEC properties due to narrow bandgaps, suitable band edge positions for oxygen evolution reactions (OER), electrical properties, and stability [53,54]. For example, BiVO_4_ is the widely explored MV in photocatalytic and PEC studies, owing this to its bandgap alignment, optical absorption, and catalytic properties [55]. Even though the unique oxygen–vanadium bond in MVs offers superior charge storage properties, their utilization as photoelectrodes has been extensively explored, owing this to their favorable optical and electrical properties. Generally, the MVs are categorized under the M_x_V_y_O_z_ structural family in which M is the metal having a lower oxidation state, whereas the V is commonly present in a wide range of oxidation states based on the crystal phase preferably being in the stable V^5+^ state [56,57]. In MVs, the presence of vanadium provides different bonding versatility, owing this to its ranging oxidation states from V^2+^, V^3+^, V^4+^, and V^5+^ states, among which V^5+^ is known to be significantly stable compared to the other states [58]. The hybridized O 2p orbital in M_x_V_y_O_z_ structures offers facile regulation of both the Fermi level and the band edge positions, allowing the tuning of optical, band edge, and catalytic properties in favor of water splitting reactions. Importantly, the stable presence of second metal elements in M_x_V_y_O_z_ crystals along with vanadium and oxygen species provides a synergistic effect that favors catalytic effects in both electrochemical and PEC applications [55].

Among the MVs, the copper vanadates are considered a unique class of materials that offer narrow bandgaps, tunable electrical properties, and stability under illuminations. The copper vanadates are generally presented using the formula Cu_x_V_y_O_z_, implicated by the possibility of stoichiometric tuning that affects optical, electrical, band edges, and catalytic properties. That is, the percentage of Cu and V in the copper vanadates could be tuned, owing this to the availability of V in the wide oxidation state and O stoichiometries. Generally, the copper vanadates are indirect bandgap materials (~2 eV) having n-type characteristics that allow the absorption of significant regions of light in the visible range, while their intrinsic properties allow the utilization as photoanodes. Interestingly, copper vanadates are the only narrow bandgap n-type oxide-based semiconductors utilized in solar water splitting reactions, whereas most of the oxide semiconductors possess wider bandgaps. Moreover, copper vanadates are found naturally in the environment as minerals and thus their abundance and extraction become beneficial in terms of availability and commercialization. Due to the unique electric and chemical properties, the copper vanadates were extensity utilized as visible light absorbing photocatalysts for energy production, dye degradation, etc., and as electrode materials in energy storage devices. Generally, in copper-based ternary oxides, or as in copper vanadates, the hybridization of O 2p and Cu 3d orbital states allow the shift of the valence band closer to the conduction band causing the optical bandgap to be reduced, which essentially allows a wider light absorption range. Specifically, the addition of VO^4−^ in the crystal Cu-based ternary oxides allows the hybridization of states to reduce the hand edge. Furthermore, such hybridization is expected to offer efficient photo-induced catalysis and stability during the prolonged irradiation in mild alkaline electrolytes.

**Figure 3:**
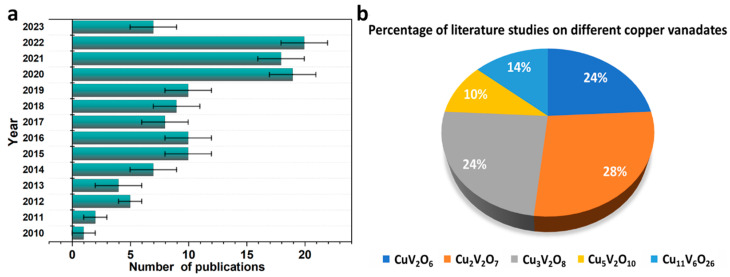
(**a**) Number of publications reported in the literature regarding the study on copper vanadates. (**b**) Percentage of published reports for specific copper vanadate stoichiometry.

A wide range of copper vanadates has been reported for different applications; however, only specific copper vanadates have demonstrated photocatalytic and photoelectrochemical water splitting capabilities. Figure 3a represents the articles published during the last decade concerning synthesis, characterization, and application studies of copper vanadate-based materials. The trend of publication indicates the rise in studies showing significant interest in copper vanadates for photocatalytic, energy storage, and sensor applications. Among the copper vanadates, only selected copper vanadates such as CuV_2_O_6_ [59,60,61,62,63,64,65,66], Cu_2_V_2_O_7_ [59,60,61,62,63,67,68,69], Cu_3_V_2_O_8_ [63,67,70,71,72,73], Cu_5_V_2_O_10_ [67,68,74], and Cu_11_V_6_O_26_ [67,68,75] have demonstrated activity toward solar water splitting reactions. Mainly, the material having a copper and vanadate (Cu:V) ratio of 1:1 (Cu_2_V_2_O_7_) has been exploited extensively, followed by CuV_2_O_8_ and Cu_3_V_2_O_8_, as shown in Figure 3b. However, there is limited literature on Cu_11_V_6_O_26_ and Cu_5_V_2_O_10_ with regard to photocatalytic activities (Figure 3b). Note that the copper vanadates have been reported only for photocatalytic/PEC oxygen productions, and have not been utilized for the hydrogen productions in the literature due to the band edge position of copper vanadate alignment with standard water redox potential that supports the OER process more favorably than the HER reaction. Nevertheless, the narrow bandgap of copper vanadates is capable of producing higher photocurrents in theoretical terms and thus is capable of producing both H_2_ and O_2_ if the photocurrents reach high enough to energize the electrons for H_2_ production. However, experimentally reaching such a high photocurrent is challenging due to several factors, including poor catalytic effect and charge transfer/diffusion properties. Such conditions are comparable to BiVO_4_-based photoanodes which allow the OER process and attain low photocurrents without any H_2_ evolution in the pristine form. However, when doped with suitable metals and supported with a catalyst, the photocurrents of BiVO_4_ reach significantly high, and both the H_2_ and O_2_ productions occur at a standard bias potential of 1.23 V vs. RHE. The aim of this review is to provide a comprehensive analysis and to conclude the limited research available and the need for aggressive research in terms of exploring a range of copper vanadates, doping strategies, and catalyst loadings that could reach higher photocurrent milestones in the future. Importantly, the stability of copper vanadates is known to be one of the promising aspects and thus with a strategy to achieve high currents, the copper vanadates could be used as one of the effective materials for the PEC water splitting reactions.

## 6. Synthesis Routes of Copper Vanadates in Solar Water Splitting

The synthesis approach is one of the crucial aspects of fabricating photoanodes that determines the morphology, stoichiometry, crystal structure, and thickness that influence the photocatalytic properties. Generally, control over stoichiometry is achieved through optimized precursor concentrations. However, to be used as a photoanode, the synthesis approach has to be optimized to deposit the desired copper vanadates on the transparent conducting substrate. This can be achieved either in a one-step process or a two-step method. In the one-step approach, the synthesis is generally carried out to deposit the copper vanadates directly on the substrate, while the two-step approach involves the synthesis of the copper vanadate followed by the deposition on the substrate via coating methods such as drop casting, dip coating, spic coating, etc. The copper vanadate synthesis schemes involve dip coating [70] (a precipitation method), followed by spin coating [71], drop casting [60], solution combustion [63], hydrothermal [64], electrospray [61], RF magnetron sputtering [67], spray pyrolysis/electrodeposition [69], etc.

## 7. Crystal Structure of Copper Vanadates

The stoichiometric combination and the arrangement of Cu, V, and O in copper vanadates offer a variety of crystal structures and phases [76]. Based on the molar ratio of the starting materials, synthesis method, synthesis condition, and annealing process, different copper vanadates could be tuned to occupy diverse crystal sites to form a variety of polymorphs [76]. Compared to the Cu and O, the V atoms stabilize in V^5+^ state, a configuration that complexes with six oxygens leading to the formation of octahedral complexes. Such oxygen- and vanadium-containing octahedra are arranged and/or connected in different structures via edge or corner sharing with other octahedra in chain and layers frameworks to form 3D structures based on the Cu metal and O ion or ions that result in aforementioned copper vanadates [58].

**Figure 4:**
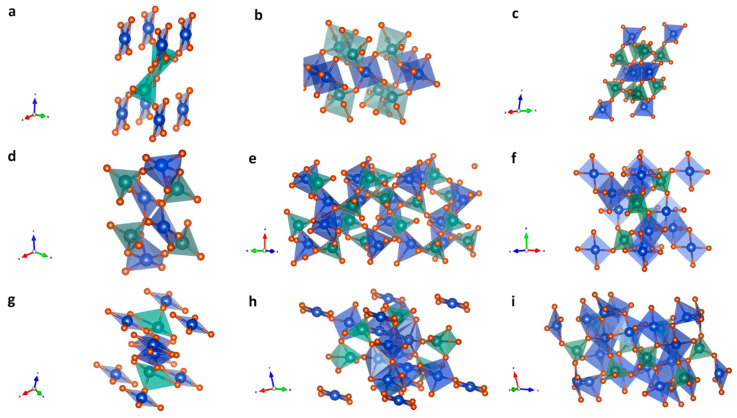
(**a**) Triclinic and (**b**) monoclinic crystal structure of CuV_2_O_6_. (**c**) Monoclinic, (**d**) triclinic, and (**e**) orthorhombic crystal structure of Cu_2_V_2_O_7_. The crystal structure of (**f**) monoclinic and (**g**) triclinic Cu_3_V_2_O_8_ phase. (**h**) The monoclinic structure of Cu_5_V_2_O_10_ and (**i**) the triclinic structure of Cu_11_V_6_O_26_.

Among the copper vanadates, CuV_2_O_6_ is generally employed in triclinic and monoclinic crystals for photocatalytic applications. The triclinic structure of CuV_2_O_6_ comprises the P̅1 space group, in which V atoms are connected to five O^2−^ atoms in a five-coordinate structure, having shorter and longer O bond lengths due to crystal distortions. The triclinic crystal contains four O^2−^ moieties attached to Cu^2+^ to form a distorted square plan structure as shown in Figure 4a. The distorted structure causes two longer and two shorter Cu-O bond lengths in the square plan structure. In the triclinic CuV_2_O_6_ structure, O^2−^ atoms appear in three different inequivalent crystal surroundings with one three-coordinate structure and two two-coordinate structures bonding. In a three-coordinate structure configuration, the O^2−^ moiety is bonded to three equivalent V^5+^ atoms in its surroundings. However, in the case of a two-coordinate structure bonding, the O^2−^ appears to be bond to both Cu^2^⁺ and V^5^⁺ atoms, one of which appears to be bonded in 150 degree bonding angles that are distorted and bent. Figure 4b represents monoclinic CuV_2_O_6_ having a C2/m space group. Similar to the triclinic structure, the V atoms are connected to five O^2−^ atoms in a five-coordinate structure, having shorter and longer O bond lengths due to crystal distortions, whereas Cu^2+^ forms a CuO_6_ octahedra via edge-sharing arrangements with four longer and two shorter bonds with O^2−^ atoms. Interestingly, the triclinic crystal of CuV_2_O_6_ creates three inequivalent O^2−^ sites in the lattice. That is, in a two-coordinate bonding configuration with both V^5^⁺ and Cu^2^⁺ atoms with a distorted bonding angle of 120 degrees. Furthermore, in a three-coordinate arrangement with three equivalent V^5^⁺ atoms, and another three-coordinate arrangement consisting of a distorted trigonal planar structure, the arrangements bond to two equivalent Cu^2+^ atoms and one V^5^⁺ atom.

The Cu_2_V_2_O_7_ is generally utilized in monoclinic, triclinic, and orthorhombic crystal phases for photocatalytic applications. In the monoclinic phase, the Cu_2_V_2_O_7_ arranges in the C2/c space group, in which both Cu^2+^ and V^5^⁺ are attached to four O^2^⁻ atoms (Figure 4c). The V^5+^ forms a VO_4_ octahedra by corner sharing the O^2^⁻ atoms, whereas the Cu^2+^ opts for a four-coordinate structure. In both Cu^2+^ and V^5+^ structures, the bond lengths attached to O^2−^ were non-equivalent due to structural distortions. In the crystal, four non-equivalent lattice sites were observed for the O^2−^ atoms. Out of these, the three non-equivalent lattice sites of O^2−^ were bonded in a two-coordinate structure, namely between two equivalent lattices of V^5+^ (linearly bonded), and between Cu^2+^ and V^5+^ with and without the distorted angle (150) arrangements in the lattice. In addition, the O^2^⁻ was noticed to be bonded to two equivalent Cu^2^⁺ atoms and one V^5+^ atom in a distorted trigonal planar arrangement. The triclinic phase of Cu_2_V_2_O_7_ forms a P̅1 space group having two inequivalent sites for Cu^2+^ and V^5+^, each, and seven inequivalent sites for the O^2^⁻ atoms in the lattice, as shown in Figure 4d. One of the Cu^2+^ forms a distorted square pyramid structure using five O^2−^ atoms that are connected to the five tetrahedra with a VO_4_ structure with a corner-sharing configuration and edge-sharing arrangements with other two Cu^2+^ equivalent square pyramidal structures. On the other hand, the second type of Cu^2+^ atoms form a distorted octahedral structure (CuO_6_) by coordinating with six O^2−^ atoms. In this, the distorted octahedra connects to the seven tetrahedra of VO_4_ and two equivalent octahedra of CuO_6_ in the surroundings via a corner-sharing and an edge-sharing configuration, respectively. The V^5+^ tetrahedra (VO_4_) forms a bond with four O^2−^ sites that connect to four equivalent octahedra and two equivalent square pyramidal structures of Cu^2+^ via corner sharing, respectively, and one V^5+^ tetrahedra (VO_4_) through corner sharing. The orthorhombic Cu_2_V_2_O_7_ stabilizes in the Fdd2 space group containing distorted CuO_5_ trigonal bipyramids and VO_4_ tetrahedra, having four inequivalent O^2^⁻ sites. In the crystal, VO_4_ tetrahedra shares one corner with another VO_4_ tetrahedra and five equivalents trigonal bipyramidal of CuO_5_. On the other hand, the CuO_5_ appears to share edges with a couple of equivalent trigonal bipyramids of CuO_5_ and corners with five equivalent VO_4_ tetrahedra.

The Cu_3_V_2_O_8_ has been exploited in both monoclinic (Figure 4f) and triclinic (Figure 4g) phases, having P2_1_/c and P̅1 space group, respectively, for photocatalytic applications. In both monoclinic and triclinic structures, V^5+^ atoms appear to form VO_4_ tetrahedra with four O^2^⁻ bonds, and both phases contain four inequivalent O^2+^ sites in the lattice. However, Cu^2+^ forms two different structures in both monoclinic and triclinic phase arrangements. That is, Cu^2+^ consists of a square coplanar structure and a square pyramidal structure with four and five O^2−^ bonds, respectively, in the monoclinic phase, whereas in triclinic structure it was bonded to four O^2^⁻ atoms to form a distorted rectangular form having a seesaw-like structure and square coplanar form. The VO_4_ tetrahedra in monoclinic Cu_3_V_2_O_8_ connects to the surrounding five CuO_5_ square pyramids (equivalent) via corner sharing. Furthermore, the distorted Cu^2+^ square pyramids share a corner with two equivalent Cu^2+^ square pyramids and five V^5+^ tetrahedra.

The monoclinic crystals of Cu_5_V_2_O_10_ form a P2_1_/c space group consisting of VO_4_ tetrahedra, CuO_5_ square pyramids, and CuO_5_ trigonal bipyramid bonded to four, five, and five O^2−^ atoms, respectively. The VO_4_ octahedra in the crystal appear to have two non-equivalent sites, one sharing the corners with two equivalent square pyramids and three equivalent trigonal bipyramids consisting of CuO_5_ structures. The second VO_4_ tetrahedra shares a corner with one trigonal bipyramid and two equivalent CuO_5_ square pyramids. Such an arrangement causes the formation of Cu^2+^ with five inequivalent sites in the lattice, such as bonding to four O^2^⁻ atoms to form distorted rectangular seesaw-like structures, distorted CuO_5_ square pyramids with five O^2^⁻ atoms, that share four and one corners with the VO_4_ tetrahedra and CuO_5_ trigonal bipyramid, respectively. Furthermore, the Cu^2+^ with four and six O^2^⁻ atoms forms a rectangular seesaw-like structure. The distorted CuO_5_ trigonal bipyramids formed by bonding to five O^2^⁻ atoms attach to four VO_4_ tetrahedra and one CuO_5_ square pyramid via corner sharing. Moreover, the atomic arrangements of both Cu and V in monoclinic Cu_5_V_2_O_10_ give rise to ten inequivalent O^2^⁻ sites. The triclinic Cu_11_V_6_O_26_ crystallizes (Figure 4i) in the P̅1 space group, having three inequivalent sites of V^5^⁺ in VO_4_ tetrahedra, six inequivalent Cu^2^⁺ sites of CuO_5_ trigonal bipyramids, a square pyramid, and CuO_6_ octahedra along with thirteen inequivalent O^2^⁻ sites.

## 8. Copper Vanadates Photoelectrodes in Water Splitting

Owing this to the suitable band edge, narrow bandgap, stability, and charge diffusion properties, copper vanadates are extensively exploited in photocatalytic applications including solar water splitting activities. The feasibility of tuning the stoichiometry of Cu and V atoms in the copper vanadates allows the exploitation of a wide range of copper vanadates with tunable properties. Hence, the utilization of a suitable synthesis method and thin film fabrication procedure dictates the morphology of the copper vanadates, while the optimized source material with synthesis conditions determines the crystal structure and stoichiometry of copper vanadates. Employing different synthesis techniques and conditions, a wide range of copper vanadates have been obtained and exploited in solar water splitting applications, as described below. Table 1 represents the photoelectrodes of copper vanadates reported along with the synthesis methods, morphologies, types of semiconducting, photocurrents, and H_2_/O_2_ gas evolution properties.

### 8.1. CuV_2_O_6_

**Figure 5:**
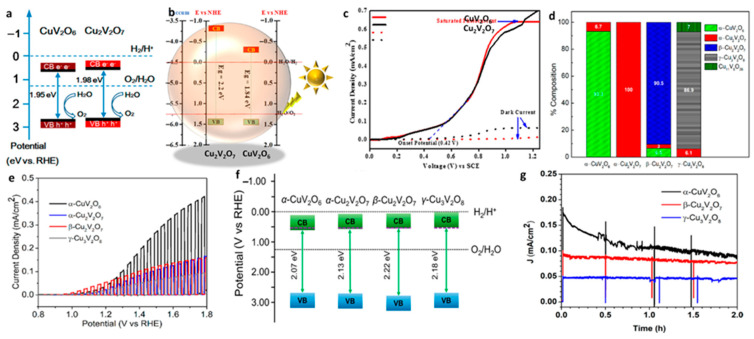
(**a**) Band edge positions of CuV_2_O_6_ and Cu_2_V_2_O_7_. Adapted with permission from [60], Copyright 2015 American Chemical Society. (**b**) Band edge positions and (**c**) photocurrents of Cu_2_V_2_O_7_ and CuV_2_O_6_ photoelectrodes. Reproduced with permission from Ref. [62], Copyright 2027, Springer Nature. (**d**) The percentage of different phases present in the synthesized copper vanadates via the solution combustion method was calculated via Rietveld refinement of XRD data. (**e**) LSV, (**f**) edge, and (**g**) stability measurements of α-CuV_2_O_6_, α- and β-Cu_2_V_2_O_7_, and γ-Cu_3_V_2_O_8_ photoelectrodes measured in borate buffer of pH 9.2. Adapted with permission from [63], Copyright 2019 American Chemical Society.

The CuV_2_O_6_ was initially explored in solar thermochemical water splitting, owing this to its active catalytic activity toward the decomposition of SO_3_ that initiates the production of oxygen in a molten state [59]. However, the first demonstration of employing CuV_2_O_6_ for the PEC water splitting reactions was reported by Guo et al. [60] in 2015. The thin films of CuV_2_O_6_ are obtained using a drop-casting method with the precursors of Cu and V with a specific quantity of ethylene glycol. The temperature of the substrate was maintained at 180 °C throughout the fabrication process followed by annealing for 2 h between the temperature of 400 and 550 °C. The drop-cast and annealed CuV_2_O_6_ possesses a triclinic phase, having particle sizes of 430 ± 50 nm interconnected to form a thin film structure of 2 µm in thickness with an indirect bandgap of ∼1.95 eV. The PEC measurements (carried out in 0.1 M borate buffer) revealed a thickness-related photocurrent, achieving the highest photocurrent of 25 µA cm^−2^ (OER photocurrent) and 220 µA cm^−2^ (in the presence of a hole scavenger) for the 2 µm thick samples. Based on the Mott–Schottky (MS) and spectroscopic measurements, the band edge positions of CuV_2_O_6_ were plotted, as shown in Figure 5a. The conduction band edge position was situated above the OER oxidation potential, whereas the valence band edge was below the OER potential, implying that the photogenerated holes are energetically suitable to carry out OER at the electrode surface. Conclusively, the prolonged exposure of CuV_2_O_6_ to AM 1.5 illuminations demonstrates excellent stability with a decrease of only 14% in photocurrent until 3 h of OER production with a rate of 4.5 µmol L^−1^ per 20 min and faradaic efficiency of 70%.

Khan et al. [62] have established a hydrothermal scheme to synthesize peculiar platelets structured as CuV_2_O_6_ of particle sizes 50–70 nm using pluronic P-123 as a surfactant. To obtain the stoichiometric CuV_2_O_6_, the hydrothermally synthesized samples are subjected to annealing at 500 °C, yielding a triclinic phase. The optical characterization revealed a bandgap of 1.84 eV with an absorption onset of 670 nm. Such a narrow bandgap is known to be well suited for PEC applications, owing this to its significant absorption range. The band edge potential of synthesized CuV_2_O_6_ was calculated using optical characterization data and theoretical calculations. As shown in Figure 5b, the VB and CB positions were determined to be below and above the standard OER and HER potential, respectively. Interestingly, the location of the CB edge at a negative potential above 0.0 V vs. RHE is contradictory to the other published results. Moreover, the PEC studies conducted in 0.5 M Na_2_SO_4_ solution of pH 7.2 produced a photocurrent of 0.64 mA cm^−2^ at 1.2 V vs. saturated calomel electrode (SCE) (Figure 5c).

During the synthesis of copper vanadates, the tuning ability of stoichiometry is essential to study the effect of all the relevant parameters that influence the PEC properties. Among the proposed synthesis approaches, the solution combustion method provides effective tuning of the stoichiometry with simple and cost-effective experimental techniques, as reported by Hossain et al. [63]. In the synthesis mixture containing Cu and V precursors (molar ratio of 0.25:0.50 M), the DL-malic acid was used as a complexing agent, which also contributes as a fuel during solution combustion carried out at ∼300 °C. As-synthesized CuV_2_O_6_ requires further annealing (550 °C) to remove its organic impurities and to crystallize in pure phases. Using the spray coating method, the CuV_2_O_6_ was deposited on substrates for PEC characterizations. The optimized and annealed CuV_2_O_6_ at 550 °C exhibited α triclinic crystal phase which was transformed to β-CuV_2_O_6_ when the annealing temperature was increased to 610 °C. Note that despite the regulation and optimization of the annealing temperature, no pure and single phase of the triclinic crystal phase of α-CuV_2_O_6_ was observed. At the optimized temperature (600 °C), a maximum of 93.3% of α-CuV_2_O_6_ was observed with an impurity of α-Cu_2_V_2_O_7_ of 6.7%, as confirmed via Rietveld refinement (Figure 5d) of the XRD data. Interestingly, the α-CuV_2_O_6_ exhibited a direct bandgap of 1.89 eV, which contradicts the previous reports that implied an indirect bandgap. For the PEC characterization, the borate buffer of pH 9.2 was used as a supporting electrolyte with solar light simulated to AM 1.5 G illumination. The α-CuV_2_O_6_ produced the highest photocurrent of 55 µA cm^−2^ at 1.23 V vs. RHE with an onset potential of ~0.8 V (Figure 5e). The band edge positions of α-CuV_2_O_6_ were evaluated using experimental and theoretical considerations and were plotted in comparison to other copper vanadates, as shown in Figure 5f. As presented, the conduction band edge and Fermi level of α-CuV_2_O_6_ were situated between 0.0 and 1.23 V vs. RHE, whereas the valence band was positioned at a significantly higher positive potential than the OER oxidation potential of 1.23 V vs. RHE (as shown in Figure 5f). The proposed band edge positions indicate that the α-CuV_2_O_6_ is effectively situated within the energy potential feasible for OER reactions from the photogenerated holes at the valence band. The I-t studies presented in Figure 5g indicate that α-CuV_2_O_6_ shows significantly lower stability than the other copper vanadates [79] due to the photo-leaching of V that causes the formation of a Cu (II) O layer on the α-CuV_2_O_6_.

**Figure 6:**
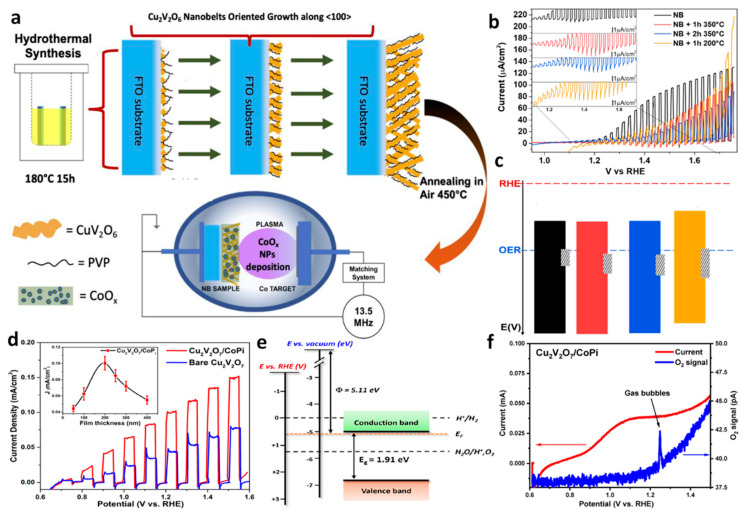
(**a**) Schematic presentation of two-step synthesis procedure of CuV_2_O_6_-CoO_x_ composite. (**b**) LSV measurements CuV_2_O_6_-CoO_x_ composite under chopped illumination. (**c**) The plotted band edge locations with respect to RHE values using MS data. The shaded region represents the cathodic spikes noticed during the measurements with an approximate potential width and position at CB and VB (**a**). Adapted with permission from [64], Copyright 2020 American Chemical Society. (**d**) LSV of β-Cu_2_V_2_O_7_ before and after the CoPi catalyst coating. (**e**) The band edge of *β*-Cu_2_V_2_O_7_ was determined using spectroscopic and electrochemical data. (**f**) PEC and O_2_ production activity of CuV_2_O_6_-CoO_x_ were measured using electrochemical mass spectroscopy. Adapted with permission from [69], Copyright 2020 American Chemical Society.

To overcome the limitation to produce pure phase α-CuV_2_O_6_, Hossain et al. [65] extended their solution combustion method to yield pure α-CuV_2_O_6_ with a modified method called the time-efficient solution combustion. To limit or to avoid the formation of α-Cu_2_V_2_O_7_ or V_2_O_5_, as an impurity in the α-CuV_2_O_6_ phase, the combustion method was strategically tuned via pH control (at 4) and NaOH washing procedure upon post-synthesis. The Rietveld refinement carried out for the XRD data confirms the presence of a single triclinic phase of pure α-CuV_2_O_6_. The pure triclinic α-CuV_2_O_6_ phase shows an indirect bandgap of 1.83 eV. The pure phase of α-CuV_2_O_6_ shows a slightly higher onset potential of 0.95 photocurrents compared to the impure phases, as discussed earlier (0.8 V). At a bias potential of 1.74 V, the photocurrent rises to 0.75 mA cm^−2^, which was double the value reported for impure α-CuV_2_O_6_. Similarly, Girardi et al. [64] have studied the effect of a catalyst coating on CuV_2_O_6_ and its influence on PEC properties. The triclinic CuV_2_O_6_ was deposited on fluorine-doped tin oxide (FTO) using hydrothermal (with polyvinylpyrrolidone as a capping agent) method followed by annealing. Using a Co source in RF sputtering, the CoO_x_ catalyst was deposited on the CuV_2_O_6_ films. Figure 6a presents the synthesis scheme of CuV_2_O_6_-CoO_x_ composite providing favorable p-n junction due to the n-type CuV_2_O_6_ and p-type CoO_x_. The hydrothermal procedure produces nanobelts arranged systematically on the substrate, having a length of 3000 nm and a width of 300 nm. Such a nanostructure offers an enhanced surface area with decreased grain boundaries and defects, which positively influence the charge separation by providing decreased recombination centers. The subsequent deposition of CoO_x_ posed no significant change in morphology. The XRD studies confirm the presence of 86% of the triclinic CuV_2_O_6_ phase along with a minor impurity phase of 14% of monoclinic β-Cu_2_V_2_O_7_. The PEC measurements (Figure 6b) indicates that the samples coated with CoO_x_ display poor photocurrent and onset potential compared to the uncoated samples, indicating negative effect of the catalyst on the CuV_2_O_6_ PEC properties. The inset in Figure 6b indicates the photocurrents showing cathodic spikes influencing the position and the potential width. Figure 6c shows the band edge positions plotted using MS plots and the shaded region shows the potential location and width caused by the cathodic spikes during PEC measurements. Importantly, the results indicate that despite coating the CuV_2_O_6_ with an effective OER catalyst, which provides favorable band edge arrangement, no significant shift in onset potential or rise in current was observed, indicating selective intrinsic properties of CuV_2_O_6_.

### 8.2. Cu_2_V_2_O_7_

The Cu_2_V_2_O_7_ material was first employed for the PEC water splitting reactions in 2015 [60]. A facile drop-casting technique was employed to produce Cu_2_V_2_O_7_ thin films, using the solutions containing Cu and V precursors dissolved in ethylene glycol followed by drying at 180 °C and annealing at 400–550 °C for 2 h. The optimized sample considered for the detailed study consists of a monoclinic phase having a thickness of 1 µm and containing interconnected particles of sizes 630 ± 50 nm. The spectroscopic data indicated an optical bandgap of 1.98 eV. The PEC measurements were carried out in 0.1 M of a borate buffer, which exhibited an OER photocurrent of 35 µA cm^−2^ and sulfite oxidation current of 120 µA cm^−2^. The band edge position of Cu_2_V_2_O_7_ presented in Figure 5a confirms the valence and conduction band edge between the OER potential indicating the suitability for solar OER reactions. Importantly, significant stability was noticed under the prolonged illumination with a decrease in the photocurrent of only 10% with a faradaic efficiency of 80%, indicating the applicability of Cu_2_V_2_O_7_ for PEC water splitting reactions.

The copper vanadate thin films could also be obtained using the electrospray coating method, as demonstrated by Kim et al. [61]. The average particle size of the electro-sprayed copper vanadates was around 100 nm with a film thickness of 2 µm. The annealing process was used to remove organic impurities. However, the films fabricated using the electrospray coating method and annealed at 600 °C yielded the mixture of CuV_2_O_6_ and Cu_2_V_2_O_7_ phases and thus showed poor PEC properties (OER photocurrent of 40 µA cm^−2^). On the other hand, the samples annealed at 500 °C showed dominant Cu_2_V_2_O_7_ that showed comparatively enhanced OER photocurrent of 100 µA cm^−2^. Furthermore, the Cu_2_V_2_O_7_ phase thin films exhibited an enhanced photocurrent of 650 at 1.23 V of bias potential vs. RHE in the presence hole scavenging sulfite electrolytes. This value was significantly higher than that of the Cu_2_V_2_O_7_ films synthesized via RF magnetron sputtering, which produced 36 µA cm^−2^ for OER, and 51 µA cm^−2^ for the sulfite oxidation reaction with an onset of 0.1 and 0.79 V vs. RHE, respectively [67]. In our previous study [68], we proposed a facile hydrothermal method to obtain the monoclinic Cu_2_V_2_O_7_ having nanoplate morphology. The morphology and the stoichiometry were specifically tuned via the control of synthesis condition including the use of urea as the capping agent. The unique morphology and single-phase characteristic offered an enhanced photocurrent value of 410 µA cm^−2^ (at 1.23 V vs. RHE), the highest reported for the Cu_2_V_2_O_7_ photoanodes. The Cu_2_V_2_O_7_ displayed n-type characteristics with a bandgap of 1.98 eV. Mainly, the band edge properties proposed in this work agree with the literature reports, indicating favorable band potential to carry out OER during water splitting.

A hydrothermal method, proposed by Khan et al. [62], is used to obtain micro-flakes structured as Cu_2_V_2_O_7_ using surfactant pluronic P-123. Specifically, the monoclinic phase of Cu_2_V_2_O_7_ resulted from annealing the as-synthesized samples at a temperature of 250 °C. The optical measurements indicated an absorption onset of 565 nm attributed to the optical bandgap of 2.2 eV. Using the data from optical measurements and theoretical calculations, the band edge positions concerning vacuum energy levels and NHE potential were determined, as presented in Figure 5b. Even though the optical properties agree with the literature reports, the band edge positions were observed to be exceptionally different, in which the CB is noted to be at a negative potential compared to water reduction potential. That is, the conduction band edge appeared to have a negative potential compared to the 0.0 V vs. NHE, which contradicts the literature reports. Furthermore, the valence band was at a higher potential compared to the water oxidation potential, which agrees with the literature data. The PEC studies showed that Cu_2_V_2_O_7_ is capable of producing nearly 0.7 mA cm^−2^ (at 1.2 V vs. SCE) of photocurrent in the neutral electrolyte of pH 7.2 (Figure 5c).

Using the solution combustion method along with an optimized annealing temperature range, both *α*-Cu_2_V_2_O_7_ and *β*-Cu_2_V_2_O_7_ phases could be obtained [63]. During the synthesis, the utilization of a molar ratio of 0.5:0.5 M of Cu and V precursors is essential to yield Cu_2_V_2_O, whereas the annealing step followed by the synthesis produces either *α*-Cu_2_V_2_O_7_ and *β*-Cu_2_V_2_O_7_ based on the temperature of annealing. The controlled annealing not only removes the organic impurities (induced via DL-malic acid fuel) but also helps to crystallize the particles. As shown in Figure 5d, annealing at 500 °C generates pure *β*-Cu_2_V_2_O_7_ that tends to transform into the *α*-Cu_2_V_2_O_7_ phase with an increase in temperature above 500 °C. As a result, a complete transformation to the *α*-Cu_2_V_2_O_7_ phase was observed at 610 °C. Unlike *α*-Cu_2_V_2_O_7_, a completely pure *β*-Cu_2_V_2_O_7_ phase was not observed, as revealed from the phase composition calculation carried out via Rietveld refinement that indicated the appearance of α-CuV_2_O_6_ (6.5%) and *α*-Cu_2_V_2_O_7_ (3%) as the impurity phases (Figure 5d) due to the limitations during the crystal transformation that were created. Both *α*-Cu_2_V_2_O_7_ and *β*-Cu_2_V_2_O_7_ exhibited indirect bandgaps of 2.13 and 2.22 eV, respectively. The *α*-Cu_2_V_2_O_7_ and *β*-Cu_2_V_2_O_7_ produced a maximum photocurrent of 0.030 and 0.065 mA cm^−2^ in the borate butter electrolytes (Figure 5e). The suitability of the band edge potential was evaluated using the PEC data and is presented in Figure 5f. The Fermi level and CB of both the *α*-Cu_2_V_2_O_7_ and *β*-Cu_2_V_2_O_7_ were situated below 0 V vs. RHE and above 1.23 V vs. RHE. On the other hand, the valence band edge was positioned at a significantly positive potential compared to 1.23 V vs. RHE. This indicates that both the *α*-Cu_2_V_2_O_7_ and *β*-Cu_2_V_2_O_7_ are capable of performing OER using solar illuminations. The stability experiments (Figure 5g) indicated that *β*-Cu_2_V_2_O_7_ shows exceptionally superior stability over a prolonged OER process.

The aforementioned discussion demonstrates that *β*-Cu_2_V_2_O_7_ suffers from serious drawbacks that limit its OER activity. The slow OER kinetic properties of *β*-Cu_2_V_2_O_7_ could be improved by utilizing a suitable catalyst, as demonstrated by Song et al. [69]. The authors employed the electrodeposition method to coat the CoPi catalyst on the β-Cu_2_V_2_O_7_ photoelectrode prepared using the spray pyrolysis method. The pristine *β*-Cu_2_V_2_O_7_ displays (at 1.23 V vs. RHE) the photocurrent of 50 µA cm^−2^ under illumination. In contrast, the catalyst supported *β*-Cu_2_V_2_O_7_ shows an increased photocurrent of 100 µA cm^−2^ at 1.23 V vs. RHE, which is double the value compared to the pristine *β*-Cu_2_V_2_O_7_ (Figure 6d). In agreement with the previous reports, the band edge position of *β*-Cu_2_V_2_O_7_ was established to be between the OER potential, as demonstrated in Figure 6e. To determine the band edge potential, the authors analyzed the data from UPS, MS, and absorbance measurements. The results corroborate with the values within the literature reported for conduction and valence band locations concerning vacuum and RHE values. As displayed in Figure 6f, the *β*-Cu_2_V_2_O_7_ photoelectrodes coated with CoPi exhibited O_2_ evolution with a faradaic efficiency of 96%. Importantly, the results of this work demonstrate that the poor OER kinetics of *β*-Cu_2_V_2_O_7_ are due to the short carrier diffusion length, which could be improved via catalyst coating. Using time-resolved microwave conductivity studies, the carrier diffusion length of *β*-Cu_2_V_2_O_7_ was determined to be ∼28 nm. Such a short carrier diffusion length is generally expected due to low carrier movement in the bulk of the material.

### 8.3. Cu_3_V_2_O_8_

**Figure 7:**
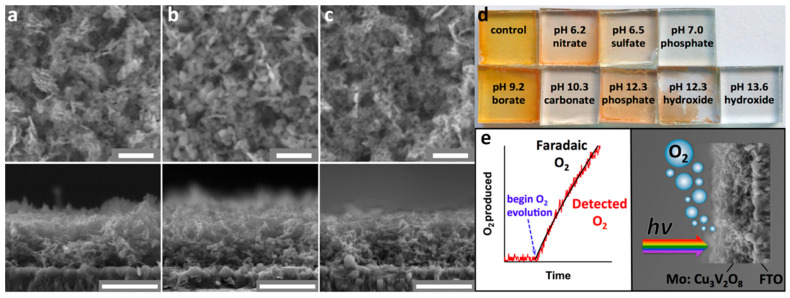
SEM (scale bar of 200 nm for surface and 500 nm for cross-section) images of (**a**) Cu_3_V_2_O_7_(OH)_2_·2H_2_O, (**b**) Cu_3_V_2_O_8_, and (**c**) Mo-doped Cu_3_V_2_O_8_. (**d**) Photographic images of Cu_3_V_2_O_8_ thin films on FTO-immersed for 48 h in different electrolytes of pH between 6.2 and 13.6, indicating stability and corrosion. (**e**) The theoretical and experimental plot of O_2_ production with solar water splitting activity presentation of Mo-doped Cu_3_V_2_O_8_ under visible light irradiation. Adapted with permission from [61], Copyright 2015 American Chemical Society.

The first report of utilizing copper vanadates for solar water splitting was demonstrated in 2015 by Jason et al. [70] with the use of Cu_3_V_2_O_8_. This report suggested that copper vanadates are the promising single-junction electrode systems for PEC water splitting, owing this to their suitable properties and thus triggering new research orientation toward material fabrication based on n-type ternary oxides having narrow bandgaps. In this report, the Cu_3_V_2_O_8_ nanoparticles were first synthesized using a facile solution-based method followed by annealing at 425 °C. The material was then systematically drop-cast onto the transparent conducting oxide surface via the dip coating method to fabricate photoelectrodes. The annealing step ensures the transformation of Cu_3_V_2_O_7_(OH)_2_·2H_2_O to Cu_3_V_2_O_8_ to provide essential crystallinity, morphology, and semiconductor properties required for photocatalytic applications. Furthermore, a change in color from bright orange to orange/brown was noticed, indicating the change in the crystal structure as well as the removal of water and organic impurities. The procedure was also exploited to fabricate the Mo-doped Cu_3_V_2_O_8_ photoelectrodes that provide enhanced efficiency compared to a pristine photoelectrode. The morphology of synthesized, annealed, and Mo-doped Cu_3_V_2_O_8_ exhibited nanoparticle structure with a porous arrangement without any significant difference upon annealing and Mo incorporation (Figure 7a–c). In addition, the thickness of the Cu_3_V_2_O_8_ films remained unchanged at ∼500 nm. The absorption measurements revealed the optical bandgap was ~2.05 eV [70], implicating its suitability as a narrow bandgap photoelectrode for visible light-assisted water splitting. Generally, the ternary vanadium oxides are known to exhibit higher stability at a limited pH range in aqueous electrolytes. This was confirmed by placing the Cu_3_V_2_O_8_ photoelectrodes in aqueous electrolytes between the pH values of 6.2 and 13.6 (Figure 7d). The digital images presented in Figure 5d indicate that Cu_3_V_2_O_8_ exhibits poor stability under all pH conditions, except in the borate buffer of pH 9.2 [70]; thus, it was employed in the electrochemical characterization and water splitting experiments. During electrochemical characterizations, a positive slope in the MS plot was observed. This confirmed the n-type properties of the Cu_3_V_2_O_8_ electrode. Based on the MS plots, the conduction band of Cu_3_V_2_O_8_ was estimated to be located between 0.65 and 0.45 V vs. RHE and thus its valence band could be situated at 2.6 V vs. RHE (based on the bandgap of 2.05 eV). The approximate band edge positions obtained via electrochemical measurements indicate the valence band of Cu_3_V_2_O_8_ is ideally placed below (more positive than) the water oxidation potential and thus confirms that the photogenerated holes are energetically capable of oxidizing water to O_2_ molecules under visible light irradiation [70]. The PEC experiments with a hole scavenger revealed that the Cu_3_V_2_O_8_ surface properties are kinetically hindered in the oxidation of H_2_O to O_2_ reaction due to the recombination at the electrolyte interface [70]. To overcome the surface kinetic limitations, a catalyst was used; however, no significant increase in the PEC activity was noticed. Interestingly, the doping of Mo produced increased photocurrent, electron diffusion length, incident photon-to-current efficiency (IPCE), and absorbed photon-to-current efficiency values with sustained production of O_2_ with a significant faradaic efficiency (Figure 7e).

Similarly, Cr doping enhanced the PEC activity of Cu_3_V_2_O_8_, as reported by Drialys et al. [71], and was synthesized using a facile aqueous precipitation approach. The synthesis protocol involves the initial step of obtaining Cu_3_V_2_O_7_(OH)_2_·2H_2_ O nanoparticles via the precipitation method followed by the addition of Triton X-100 to a specific amount of the product to yield an effective dispersion solution that allows the uniform deposition on the FTO substrate. During this step, the desired amount of Cr precursors could be introduced to obtain the doped copper vanadate followed by spin coating the FTO substrate. The final step of the fabrication involves the annealing step at 425 °C for 1 h to dehydrate the samples to obtain pure crystalline Cu_3_V_2_O_7_. Before the annealing, the samples were preheated at 200 °C for 2 min to ensure effective deposition. Together with the deposition method, the proposed synthesis method allowed the facile tuning of doping density and thickness control, indicating the versatility of this method. The thickness of the Cu_3_V_2_O_7_ was controlled by the spin coating steps. As-synthesized hydrated copper vanadates exhibited nanoflake morphology sizes between 70 and 80 nm, which were transformed to a globular nanostructure after the annealing process, having ~40 to 100 nm in particle sizes. Interestingly, the particle sizes appeared to decrease after the Cr doping from around 20 to 80 nm. The crystal structure of the synthesized Cu_3_V_2_O_8_ was a monoclinic phase having a P21/c space group. The absorbance measurements indicated the indirect bandgap of 2.0 eV with no significant change, even after the Cr doping. The PEC measurements conducted in a borate buffer of pH 9.2 indicated a maximum photocurrent of 66 µA cm^−2^ for the Cu_3_V_2_O_7_ doped optimally at 0.75%. In the presence of a hole scavenger (sulfite), the photocurrent increased to 100 µA cm^−2^. The MS measurements confirmed the positive slope indicating the n-type properties, whereas the flat band potential approximated the position of CB and VB edge positions. These observations indicated that the band edge position of Cu_3_V_2_O_8_ is ideally situated for the facile OER process under the simulated solar illumination. The doped samples exhibited a photostability of 1 h with an O_2_ production rate of 1.5 µmol cm^−2^ and an increased faradaic efficiency of 95%. Importantly, the theoretical calculations carried out using the bandgap and absorbance characteristics indicated the capability of Cu_3_V_2_O_8_ to produce as high as ~12 mA cm^−2^ of photocurrent with a bandgap of 2.0 eV. However, due to several limitations, achieving high photocurrent is challenging. Conclusively, the results implicate that doping is an effective strategy that provides improved charge separation with decreased recombination in Cu_3_V_2_O_8_ photoanodes. Interestingly, another report [73], using triclinic Cu_3_V_2_O_8_ doped with 2% of Cr exhibited HER activity. Even though the authors did not provide significant evidence regarding the locations of conduction and valence band edge concerning overall water splitting potential, an impressive H_2_ production with a rate of 288 μmol h^−1^ g^−1^ was recorded. The evidence of H_2_ evolution indicates that Cu_3_V_2_O_8_ could be an ideal candidate for PEC water splitting and thus relevant research and data are essential for its effective and efficient utilization.

**Figure 8:**
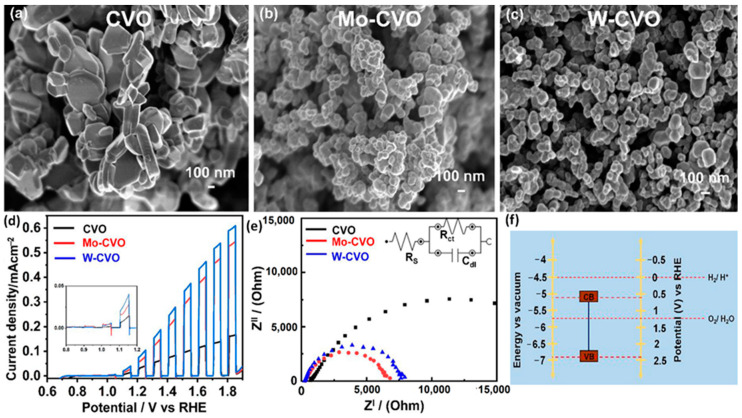
The SEM image of (**a**) undoped, (**b**) Mo-doped, and (**c**) W-doped Cu_3_V_2_O_8_. (**d**) Photocurrents (recorded under chopped illumination) and (**e**) Nyquist plots (applied bias of 0 V vs. RHE) for undoped, Mo-doped, and W-doped Cu_3_V_2_O_8_ in borate buffer solution (pH—9) under illumination. Inset in (**d**) shows the onset potential. (**f**) Band edge diagram of Cu_3_V_2_O_8_ plotted with respect to vacuum energy and potential vs. RHE compared to overall water splitting potentials. Adapted with permission from [63], Copyright 2020 American Chemical Society.

The doping of W and Mo to Cu_3_V_2_O_8_ [72] is known to offer improved PEC properties compared to the Cr dopant. Pulipaka et al. [72] have proposed a precipitation method to synthesize Mo- and W-doped Cu_3_V_2_O_8_ followed by spin coating these molecules onto a transparent conducting substrate followed by annealing. The precipitated and deposited Cu_3_V_2_O_8_ showed a monoclinic crystal phase having nanoparticle morphology of ~300 nm of particle size (Figure 8a). The presence of W (Figure 8b) and Mo (Figure 8c) dopants interacts with the grain boundaries of the particles during the synthesis process as a result of decreased energy of formation; thus, stabilizing the particle sizes to ~70 nm, as shown in Figure 6c and Figure 8b. Similar to the Cr, the doping of Mo and W does not cause any significant change in the optical bandgap. The PEC measurements conducted in borate buffer of pH 9 indicated enhanced photocurrents after the Mo and W doping. At an applied bias of 1.85 V vs. RHE under the illumination, the undoped Cu_3_V_2_O_8_ exhibited a photocurrent of 0.18 mA cm^−2^, which increased to 0.60 and 0.55 mA cm^−2^ upon W and Mo doping, respectively, with a cathodic shift in the onset potential (Figure 8d). Furthermore, the doping of W and Mo in Cu_3_V_2_O_8_ caused an increased carrier density that offered an increase in the majority of carriers for improved conductivity. Hence, the doping of Mo and W ensures suppression in the recombination by providing effective separation of photogenerated charges in Cu_3_V_2_O_8_. Such a positive impact on the PEC properties in Cu_3_V_2_O_8_ is noticed alongside an optimized Mo and W of 2%. The Nyquist plots presented in Figure 8e confirm decreased charge diffusion resistance at both bulk and electrolyte interface of Cu_3_V_2_O_8_ upon Mo and W doping. The band edge studies performed via MS studies (Figure 8f) confirm a shift in the flat band to a negative direction, indicating an upward shift in the CB. Importantly, the band edge positions of Cu_3_V_2_O_8_ plotted based on the experimental data indicate that the CB and VB edge positions are situated at an ideal position to carry out facile OER activity using photogenerated charges.

**Figure 9:**
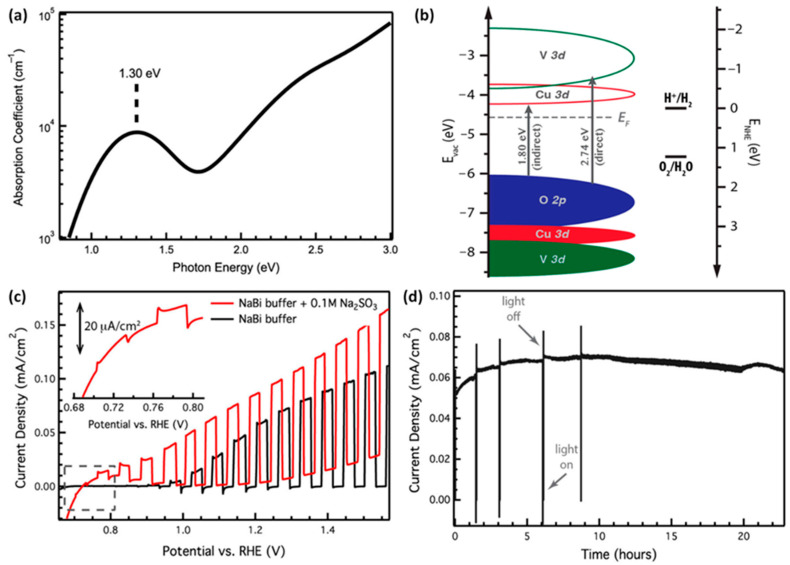
(**a**) Absorption coefficient plot as a function of the photon energy of γ-Cu_3_V_2_O_8_. (**b**) The band edge electronic structure of γ-Cu_3_V_2_O_8_ shows indirect and direct transition pathways. (**c**) LSV (red line indicates measurements under sulfite electrolyte) and (**d**) I-t plot of γ-Cu_3_V_2_O_8_ measured under AM 1.5 G illumination using pH 9.2 borate buffer electrolyte. Inset in (**c**) shows the magnified image of the voltammogram, indicating onset potential. Adapted with permission from [77], Copyright 2017 American Chemical Society.

The potential advantages of γ-Cu_3_V_2_O_8_ as a photoelectrode and its electronic band edge transition mechanism have been reported by Jian et al. [77]. The optical characterization study (absorption/transmittance) indicates the presence of an indirect bandgap in γ-Cu_3_V_2_O_8_ of 1.80 eV, a value closer to the previous reports. However, the absorption coefficient results in the sub-bandgap region implicate the presence of a weak absorption peak at 1.30 eV (Figure 9a), confirming the existence of a unique transition mechanism. The appearance of such a peak was attributed to the localized-on-site ligand field excitation (generally observed in *d*^9^ configuration) due to Cu (II) states, which are ineffective in producing essential charge carriers to participate in routine material conductions. The resonant inelastic X-ray, XPS, and X-ray absorption spectroscopy measurements confirm the dominance in the band edge with the presence of significant hybridization between the O 2p states and valence bands (Figure 9b). This study indicates that the conduction band minimum mainly contains unoccupied Cu 3d levels, whereas the valence band maximum constitutes the characteristics of O 2p states, as depicted in Figure 9b. The presence of a dominant O 2p in the VB maximum tends to bury the V 3d and Cu 3d states below the energy orbitals. Similarly, the CB minimum constitutes unoccupied orbitals of Cu 3d states followed by V 3d states above it at higher states within the band, as shown in Figure 9b. Such a band structure is classified as a charge transfer insulation property of γ-Cu_3_V_2_O_8_. The orbital distributions indicate the presence of an indirect bandgap energy of 1.80 eV arising from the transition of the O 2p → Cu 3d states. On the other hand, O 2p → V 3d transitions could also arise, leading to an indirect transition energy of 2.74 eV. The AM 1.5 G illumination on γ-Cu_3_V_2_O_8_ produces the OER photocurrent value of 62 μA cm^−2^, which increases to 91 μA cm^−2^ with a cathodic shift in the onset in the presence of an electron donor electrolyte (Figure 9c). Importantly, the I-t plots (Figure 9d) demonstrate superior and extended stability of the photocurrent under solar OER conditions up to 20 h, indicating the exceptional photostability of γ-Cu_3_V_2_O_8_ compared to standard photoanodes.

The utilization of the radio frequency (RF) magnetron co-sputtering method provides an effective method for copper vanadate synthesis with tunable stoichiometry, as demonstrated by Jiang et al. [67]. In this report, several copper vanadates were synthesized [67], among which γ-Cu_3_V_2_O_8_ was obtained by tuning the sputter condition using Cu and V metal targets. For the deposition, 400 °C of target temperature is recommended. With the control over sputtering time, the γ-Cu_3_V_2_O_8_ films of about 300 nm in thickness could be fabricated and followed by annealing at 550 °C. The γ-Cu_3_V_2_O_8_ films exhibited a thin film appearance with a homogeneous arrangement of particle sizes between 150 and 300 nm with significantly low surface porosity. Interestingly, the γ-Cu_3_V_2_O_8_ films showed a bandgap of 1.95 eV, which was lower than the films obtained using precipitation methods. The PEC measurements performed under the AM 1.5 G illumination in borate buffer of pH 9.3 exhibited a photocurrent of 71 µA cm^−2^ with an onset potential of 0.94 V vs. RHE. The photocurrent showed significant enhancement (105 µA cm^−2^) with a cathodic shift in onset potential to 0.78 V in the presence of an electron donor electrolyte. Even though the photocurrent presented in this report is lower than in the previous reports, the lower bandgap indicates that the optical properties could be tuned with the synthesis procedure. The solution combustion method also provides facile tuning of the stoichiometry during the synthesis of copper vanadates, as demonstrated in the study by Hossain et al. [63]. The procedure involves the use of DL-malic acid as fuel and a complexing agent along with Cu and V precursors. The solution combustion is performed at ∼300 °C in a muffle furnace for dehydration, followed by ignition. After the solution combustion step, the films are subjected to annealing to eliminate the organic impurities and to produce a pure crystalline phase. The thin films for the PEC measurements are fabricated using the spray coating method, and a borate buffer of pH 9.2 is used as a supporting electrolyte. Interestingly, the authors report a slightly wider bang gap of 2.28 eV compared to the literature reports. This could be attributed to the synthesis process, particle size, and mainly due to the crystal phases. A detailed XRD study carried out with respect to the annealing conditions has revealed that the γ-Cu_3_V_2_O_8_ crystals contain some minor impurities of other phases (α-Cu_2_V_2_O_7_ and Cu_11_V_6_O_26_) and thus are not found in a purely 100% phase, as shown in Figure 5d [77]. Under the AM 1.5 G illumination, the γ-Cu_3_V_2_O_8_ exhibited a photocurrent of 25 µA cm^−2^ (Figure 5e). Even though the photocurrents that are generated are slightly lower than the reported ones, the authors studied several crucial insights into the γ-Cu_3_V_2_O_8_ material. Specifically, γ-Cu_3_V_2_O_8_ offers suitable valence band edge positions (Figure 5f) concerning the OER potential with the conduction band edge situated at 0.66 V vs. RHE, which is in precise agreement with the previous reports and shows negative photovoltage that indicates n-type characteristics which experience poor OER kinetics at the electrolyte interface. Furthermore, the stability experiments (Figure 5g) conducted under AM 1.5 G illumination confirm the superior stability of γ-Cu_3_V_2_O_8_ electrodes.

### 8.4. Cu_5_V_2_O_10_

The first report of using Cu_5_V_2_O_10_ for the PEC water splitting application was demonstrated in 2018 by Jian et al. [67]. The authors used the RF magnetron co-sputtering method to tune the desired stoichiometry of copper vanadates at 400 °C followed by post-synthesis annealing at 550 °C. As-synthesized Cu_5_V_2_O_10_ thin films displayed a thin film structure of 300 nm of thickness with exceptional uniformity. Such a morphology with low roughness at the surface offers significantly decreased scattering incident light which improves PEC properties. The optical measurements indicated a bandgap of 2.03 eV that exhibited a significantly low photocurrent of 1 µA cm^−2^ with a 1.34 V vs. RHE of onset potential, while the photocurrent showed enhancements to 206 µA cm^−2^ with a cathodic shift in the onset potential to 0.67 V in the presence of an electron donor (AM 1.5 G light source in borate buffer of pH 9.3). Note that the photocurrent observed for Cu_5_V_2_O_10_ is significantly low compared to other copper vanadates due to the poor charge diffusion and surface kinetics in the OER process. Importantly, the discussion concludes that optical absorption depends on the Cu and V ratio, and that the copper vanadates have a higher Cu content which indicates a stronger level of absorption; thus, they provide a higher efficiency for the charge separation process. On the contrary, the OER catalytic properties tend to be suppressed with increasing Cu content in the copper vanadates, owing this to increased recombination of charges at the surface states concerning Cu sites.

**Figure 10:**
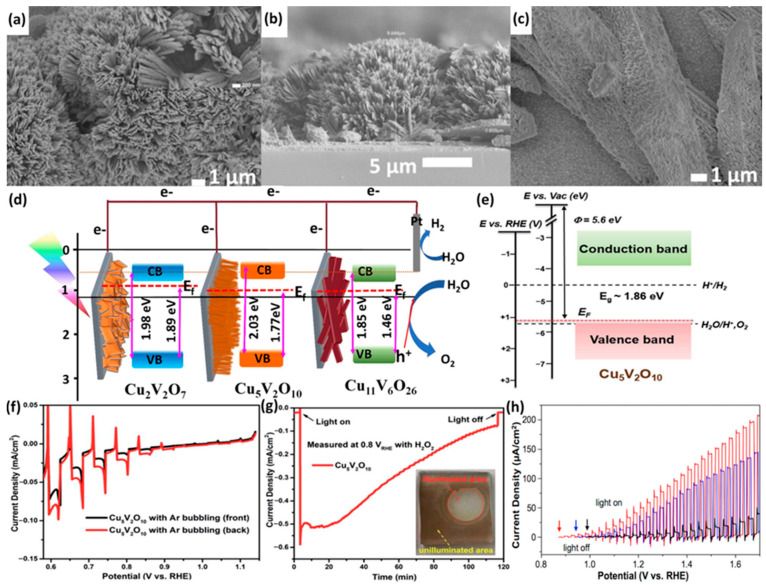
SEM images with (**a**) surface, (**b**) cross-sectional view of Cu_5_V_2_O_10_, and (**c**) SEM image of Cu_11_V_6_O_26_ synthesized via hydrothermal method. (**d**) Band locations of Cu_2_V_2_O_7_, Cu_5_V_2_O_10_, and Cu_11_V_6_O_26_ indicate n-type behavior. Copyright (2019) with permission from Elsevier [68]. (**e**) Band edge of p-type Cu_5_V_2_O_10_ plotted using MS, UPS, and optical data. (**f**) LSV and (**g**) I-t plot of p-type Cu_5_V_2_O_10_ under AM 1.5 G light source. Reproduced from Ref. [74] with permission from the Royal Society of Chemistry. (**h**) Chopped LSV plot of undoped and W and Mo doped Cu_11_V_6_O_26_. Adapted with permission from [75], Copyright 2017 American Chemical Society.

In our previous report, we proposed a facile hydrothermal method to coat Cu_5_V_2_O_10_ directly on the substrate using urea as the structure directing agent to [68]. The unique synthesis method adopted in this report produces a nanorod nanostructure arranged in a grass-like form (Figure 10a). The nanorod length was observed to be 1 μm with a rod width of 150 nm forming a thick film (~7 to 10 μm) on the substrate (Figure 10b). The Cu_5_V_2_O_10_ displayed a monoclinic phase with a bandgap of 2.03 eV. Owing this to its 1-D form with a systematic arrangement, the Cu_5_V_2_O_10_ produces a photocurrent (at 1.23 V vs. RHE) of 270 µA cm^−2^. The MS and XPS measurements indicated n-type behavior of Cu_5_V_2_O_10_ with conduction band edge and Fermi location between the water redox potentials (0 and 1.23 V vs. RHE), while the valence band edge was at a higher positive potential than 1.23 V vs. RHE (Figure 10d). Recently, p-type Cu_5_V_2_O_10_ has been reported [74]. The synthesis approach opted to obtain p-type Cu_5_V_2_O_10_ following the spray pyrolysis method with subsequent annealing in the air for 4 h at 550 °C to transform the molecule from an amorphous to a crystalline monoclinic phase having a P21/c space group. Even though there were some underlying similarities between the synthesis approach and the pure crystal phase, the core strategy of the obtained p-type behavior was not discussed. Cu5V2O10 is generally found as an n-type; hence, it is employed as a photoanode. The spray pyrolysis approach produces a thin film structure consisting of the Cu_5_V_2_O_10_ on the substrate packed closely with nanoparticle sizes of 100 to 500 nm in diameter without creating any significant porosity. The optimized film possessed a thickness of ~250 nm with significant uniformity on the substrate. The p-type Cu_5_V_2_O_10_ thin films exhibited an indirect bandgap of 1.9 eV and a direct bandgap of 2.1 eV. The time-resolved microwave conductivity results confirm the p-type Cu_5_V_2_O_10_ behavior with a charge mobility value of 2.3 × 10^−5^ cm^2^ V^−1^ s^−1^. This study further confirms that p-type Cu_5_V_2_O_10_ exhibits wavelength-dependent behavior due to the electron transition between d-d states that inhibits the production of mobile carriers. The MS measurements of Cu_5_V_2_O_10_ at different frequency values yield negative slopes, confirming p-type behavior. Furthermore, the intercept of the MS predicted the valence band edge and a 5 × 10^19^ cm^−3^ carrier density. Using the UPS, MS, and absorbance data, the band edge of the Cu_5_V_2_O_10_ molecule was plotted, as shown in Figure 10e, considering the OER and HER potential and vacuum energy values. The band diagram locations confirm that the VB is placed near the water oxidation potential along with the Fermi level. The data obtained from the MS plot along with the location Fermi level confirm the p-type characteristics of Cu_5_V_2_O_10_. The LSV data under chopped illumination (front and back) indicate a negative photocurrent reaching 20 µA cm^−2^ with an onset potential of 1.1 V vs. RHE (Figure 10f). The photocurrent spikes observed in the LSV confirm the trapping of electrons at the surface of Cu_5_V_2_O_10_, followed by recombination. Furthermore, photocurrents show a significant increase in the presence of the H_2_O_2_ electron scavenger. The I-t measurements (Figure 10g) confirm that Cu_5_V_2_O_10_ photocathodes show poor stability leading to a decrease in the photocurrent of about 85% during the 10 min of illuminations. The inset images show the leaching of Cu_5_V_2_O_10_ from the substrate.

### 8.5. Cu_11_V_6_O_26_

Jian et al. [67] have proposed an RF magnetron co-sputtering method for the synthesis of Cu_11_V_6_O_26_ thin films. The deposition approach involves the utilization of Cu and V targets sputtered to a substrate maintained at 400 °C. As-deposited films are annealed at 550 °C to transform into a crystalline phase. The Cu_11_V_6_O_26_ crystals on the substrate appear to form a thin film morphology having a thickness of 300 nm. Moreover, the films exhibit high uniformity, offering low surface roughness that could lead to the limited scattering of incident light that positively influences the PEC activity. The Cu_11_V_6_O_26_ displays a bandgap of 1.83 eV, which was noted to be significantly lower than the copper vanadates containing higher Cu content. Furthermore, the optical measurements confirm that the presence of higher Cu content allows stronger absorption ability compared to the lower Cu content copper vanadates. The PEC measurements produced a photocurrent of 53 µA cm^−2^ with an onset potential of 0.94 V vs. RHE, which shows a significant enhancement to 125 µA cm^−2^ with a cathodic shift in onset potential to 0.73 V in the presence of a sacrificial electron donor.

The stoichiometry of copper vanadates could be effectively tuned via the hydrothermal method to yield Cu_11_V_6_O_26_, as demonstrated in our previous study [68]. To yield Cu_11_V_6_O_26_, the copper precursor quantity was increased four times (molar ratio) and compared to the vanadium precursor along with the capping agent “urea”. Figure 10c shows the morphology of Cu_11_V_6_O_26_, displaying micropillar structures that are randomly arranged to form a film thickness of ~10 to 50 μm of the substrate. Even though the Cu_11_V_6_O_26_ structure shows the majority phase in the thin films, a minor presence of Cu_3_VO_4_ and CuO was also observed. The presence of multiple phases leads to a narrow optical bandgap energy of 1.85 eV. In the presence of multiple phases, with a major Cu_11_V_6_O_26_ phase and other minor phases, the thin films produced a photocurrent (at 1.23 V vs. RHE) of 76 µA cm^−2^ under AM 1.5 G illumination. The band edge positions of Cu_11_V_6_O_26_ were determined using electrochemical and spectroscopic data and are presented in Figure 10d. Importantly, the CB and edge and Fermi levels of Cu_11_V_6_O_26_ were located at a higher potential than 0 V vs. RHE. On the other hand, the valence band edge was located at a higher potential than the 1.23 V vs. RHE, indicating the feasibility of photogenerated holes to take part in the OER process.

The PEC activity of Cu_11_V_6_O_26_ could be effectively enhanced by Mo and W doping, as demonstrated by Lumley et al. [75]. Cu_11_V_6_O_26_ experiences poor OER kinetics; hence, doping becomes essential to enhance the PEC properties. The authors proposed a two-step method to fabricate both undoped and Mo/W doped Cu_11_V_6_O_26_ thin films. The initial step of synthesis involves the electrodeposition of CuO on the FTO substrate followed by the conversion of CuO into Cu_11_V_6_O_26_ or doped Cu_11_V_6_O_26_. The doping and the conversion to vanadates were achieved by coating the solution of vanadium precursors (with or without Mo and W precursor) followed by annealing at 600 °C, with an exceptionally slow ramp of 1 °C per 1 min to convert CuO to Cu_11_V_6_O_26_. During the synthesis, the washing of electrodes with NaOH becomes essential to remove unreacted residue and unwanted vanadium oxides formed during the annealing step. The washing under highly basic conditions ensures the removal of α- and β-Cu_2_V_2_O_7_. The Cu_11_V_6_O_26_ thin film on FTO forms a globular network of microparticles distributed uniformly on the substrate. The XRD pattern of Cu_11_V_6_O_26_ displays a triclinic structure, which has low symmetry with a space group of P̅1. Importantly, the doping of Mo and W displaced V atoms from the lattice leading to the shift in the XRD peak toward low theta degree values. The bandgap measurements and MS plots confirm the n-type characteristics. Importantly, the results of this work confirm that the CB edge is located at a positive potential rather than a HER potential, specifically, a more positive potential than that of the BiVO_4_ CB. The results postulate that the decrease in the bandgap of copper vanadates is generally achieved by lowering the conduction band rather than raising the valence band; thus, the band edge alignment shifts the CB and VB edge toward the OER potential. The PEC measurements indicate that the Cu_11_V_6_O_26_ produces a photocurrent of 0.010 mA cm^−2^ at 1.23 V vs. RHE, which increased to 0.035 and 0.075 mA cm^−2^ after the Mo and W doping, respectively. Importantly, the stability tests indicated that the undoped and W and Mo-doped Cu_11_V_6_O_26_ samples exhibited exceptionally high stability under solar OER conditions. Moreover, ~95% of the Faradaic efficiency of the O_2_ production was recorded. A further increase in photocurrent was observed in the presence of a hole scavenger, indicating the effective separation of photogenerated charges in the doped samples.

## 9. Conclusions and Outlooks

In summary, our discussion provides a comprehensive analysis of copper vanadates employed in PEC water splitting applications. Copper vanadates offer themselves as the alternate and more promising material option compared to the currently established binary oxide materials in solar water splitting. Hence, copper vanadates are considered a unique class of materials that offer narrow bandgaps, tunable electrical properties, and superior stability under illumination.

The copper vanadates are generally presented using the formula Cu_x_V_y_O_z_, indicating the freedom of stoichiometric tuning through which the optical, electrical, band edge, and catalytic properties could be engineered. This is due to the availability of V in a wide oxidation state and O stoichiometries. Among the copper vanadates, only specific stoichiometric polymorphs have been employed in PEC water splitting, including CuV_2_O_6_, Cu_2_V_2_O_7_, Cu_3_V_2_O_8_, Cu_5_V_2_O_10_, and Cu_11_V_6_O_26_. Given this, this review provides a comprehensive description of the advancements in cutting-edge synthesis methods and thin film fabrication methods that enable the effective tuning of morphologies, stoichiometry, and doping.

All the copper vanadates exhibit narrow bandgaps with both direct and indirect transition mechanisms, indicating the efficient utilization of visible light. Furthermore, the copper vanadates are mainly employed as n-type semiconductors due to having well-suited conduction and valence band edge positions with regard to the OER potentials; thus, this indicates the effectiveness of the photogenerated charges in copper vanadates to drive the OER activity. However, copper vanadates still require several innovative developments and modifications to improve the PEC activity, such as doping, catalyst coating, and structural tuning. Interestingly, optical absorption is dependent on the ratio of Cu and V, and the copper vanadates have a higher Cu content that enables stronger absorption and thus provides higher efficiency for the charge separation process. On the contrary, the OER catalytic properties tend to be suppressed with increasing Cu content in the copper vanadates, owing this to the increased recombination of charges at the surface states concerning Cu sites. Such behavior concerning the Cu and V ratio could be attributed to the extrinsic defect-related effects or the electronic structure that influences the intrinsic properties. Importantly, the Cu-rich copper vanadates exhibited increased surface states along with Fermi level pinning, which is known to increase the recombination at the surface and also decreases the OER efficiency. Hence, the tuning of V and Cu stoichiometry becomes important to deliver effective PEC activity.

Among the proposed strategies, catalyst coating was observed to provide both positive and negative outcomes; thus, a careful selection of catalyst and coating methods is essential. On the contrary, doping is a very effective approach to enhance the PEC activity of copper vanadates. Specifically, doping causes increased carrier density with facile charge transfer at the interface along with altered band edge positions. Unlike in BiVO_4_-based ternary oxides, the decreased bandgap in copper vanadates and the band edge shift upon doping is caused by the upward shift of the VB edge instead of the downward shift in the CB. Overall, this review encompasses both micro and macro perspectives, and focuses on the ongoing research and development efforts to optimize the performance and stability of copper vanadate materials in solar water splitting. Emphasis is placed on effective synthesis/fabrication methods for effective stoichiometry tuning. Furthermore, our comprehensive description of copper vanadates could open the door to new research directions for the development highly efficient and cost-effective copper vanadates for oxygen and hydrogen production. Importantly, the valuable insights discussed here offer comprehensive guidelines to design future copper vanadates with engineered properties.

## Figures and Tables

**Table 1:** The range of copper vanadates reported using different methods of synthesis, morphology, and PEC characterization results.

Material	Synthesis Method	Morphology	n-or p-Type	Photocurrent cm^−2^ (1.23 V vs. RHE)	O_2_ Production	Ref
CuV_2_O_6_	Drop-casting	Nanoparticles	n-type	~25 µA	4.5 µmol L^−1^	[60]
CuV_2_O_6_	Hydrothermal	Peculiar platelets	n-type	~0.64 mA (1.2 V vs. SCE)	NA	[62]
α-CuV_2_O_6_	Solution combustion	Nanoparticles	n-type	~55 µA	N/A	[63]
CuV_2_O_7_-CoO_x_	hydrothermal	Nanobelts	n-type	∼18 μA	N/A	[64]
α-CuV_2_O_6_	Solution combustion	N/A	n-type	∼750 μA (1.74 V vs. RHE)	N/A	[65]
Cu_2_V_2_O_7_	Drop-casting	Nanoparticles	n-type	~35 μA	~5 µmol L^−1^	[60]
Cu_2_V_2_O_7_	Electrospray	Nanoparticles	n-type	~0.1 mA	N/A	[61]
Cu_2_V_2_O_7_	Hydrothermal	Micro-flakes	n-type	~0.70 mA (1.2 V vs. SCE)	N/A	[62]
*α*-Cu_2_V_2_O_7_	Solution combustion	Nanoparticles	n-type	~30 μA	N/A	[63]
*β*-Cu_2_V_2_O_7_	Solution combustion	Nanoparticles	n-type	~65 μA	N/A	[63]
Cu_2_V_2_O_7_	RF magnetron sputtering	Nanograins	n-type	~36 µA	N/A	[67]
Cu_2_V_2_O_7_	Hydrothermal	Nanoplate	n-type	~0.41 mA	~5.8 µmol L^−1^ h^−1^	[68]
β-Cu_2_V_2_O_7_ /Co-Pi	Spray pyrolysis/Electrodeposition	Spherical particles	n-type	100 µA	45 pA	[69]
γ-Cu_3_V_2_O_8_	Solution combustion/spray coating	Nanoparticles	n-type	25 µA	N/A	[63]
γ-Cu_3_V_2_O_8_	RF co-sputtering	Thin film	n-type	71 µA (0.94 V vs. RHE)	N/A	[67]
Cu_3_V_2_O_8_ Mo-doped	Solution-based drop-casting	Nanoparticles	n-type	~20 µA and ~25 µA	~0.5 µmol cm^−2^ per 5 min	[70]
Cr/Cu_3_V_2_O_8_	Precipitation method	Nanoflakes	n-type	~66 µA	~1.5 µmol cm^−2^	[71]
Cu_3_V_2_O_8_ Mo doped W doped	Precipitation method	Nanoparticles	n-type	~0.18 mA ∼0.55 mA ∼0.60 mA (1.85 V vs. RHE)	N/A	[72]
Cr doped Cu_3_V_2_O_8_	Hydrothermal	Nanoparticle	N/A	N/A	H_2_:288 µm mol hg^−1^	[73]
γ-Cu_3_V_2_O_8_	Reactive Co-sputtering	Nanoparticle/thin film	n-type	~62 μA	N/A	[77]
γ-Cu_3_V_2_O_8_	RF co-sputtering	Thin film	n-type	~1 µA (1.34 V vs. RHE)	N/A	[67]
Cu_5_V_2_O_10_	Hydrothermal	Nanorod	n-type	~270 µA	2 µmol L^−1^	[68]
Cu_5_V_2_O_10_	Spray pyrolysis	Nanoparticles	p-type	~0.52 mA (0.8 V vs. RHE)	N/A	[74]
Cu_11_V_6_O_26_	RF magnetron co-sputtering	Thin film	n-type	~53 µA (0.94 V vs. RHE)	N/A	[67]
Cu_11_V_6_O_26_	Hydrothermal	Micropillar	n-type	~0.076 mA	1 µmol L^−1^	[68]
Cu_11_V_6_O_26_ W doped Mo doped	Electrodeposition/drop casting	Globular network of microparticles	n-type	~0.010 mA ~0.035 mA ~0.075 mA	~0.25 µmol cm^−2^ h^−1^	[75]
Cu_11_V_6_O_26_ γ-Cu_3_V_2_O_8_	Spray pyrolysis RF co-sputtering	Nanoparticles Thin film	n-type	~0.16 mA	N/A	[78]
			n-type	~1 µA (1.34 V vs. RHE)	N/A	[67]

## Data Availability

No new data were created or analyzed in this study. Data availability/sharing is not applicable to this article.
